# Degenerative Atypia in Clival Chordoma: Two Case Reports

**DOI:** 10.7759/cureus.66197

**Published:** 2024-08-05

**Authors:** Martha Lilia Tena Suck, Eliezer Villanueva-Castro, Marco Antonio Munuzuri-Camacho, Rebeca Hernández Reséndiz, Samuel Ismael Juárez-Cruz, Oriana Carolina Garcia-Diaz, Jose Alfredo Castro-Ibanez

**Affiliations:** 1 Department of Neuropathology, Instituto Nacional de Neurología y Neurocirugía Manuel Velasco Suárez, Mexico City, MEX; 2 Department of Neurosurgery, Instituto Nacional de Neurología y Neurocirugía Manuel Velasco Suárez, Mexico City, MEX; 3 Neurological Center, Centro Médico ABC, Mexico City, MEX; 4 Department of Pathology, Hospital General 450, Durango, MEX; 5 Department of Neuroimaging, Instituto Nacional de Neurología y Neurocirugía Manuel Velasco Suárez, Mexico City, MEX

**Keywords:** post-radiation changes, poorly differentiated chordoma, degenerative changes, cellular atypia, chordomas

## Abstract

In this study, we report surgical management combined with radiotherapy in two patients with typical chordoma. Different types of radiation have varied effects on chordomas when they are radiated. Classical cases display cellular atypia and fibrosis following irradiation, while necrosis and fibrosclerosis are observed after carbon ion therapy, implying that it is possible to control the tumor more effectively using carbon ion therapy with minimal side effects.

## Introduction

Chordoma was described by Rudolf Virchow in 1846 when he first detected its occurrence on a dorsum sellae; he coined the term "chordomata" [1]. A mesoderm-derived structure involved in neurulation and embryonic development, the embryonic notochordal vestiges of the axial skeleton give rise to uncommon, aggressive, low-grade malignant solid tumors called choreomas, which are formed from the bone. The notochordal cells have large intracytoplasmic vacuoles and are surrounded by an acellular notochord sheath rich in collagens, laminins, and proteoglycans [2].

Chordomas are rare invasive bone tumors that may occur anywhere along the neuraxis, always arising in the axial skeleton, including the skull base and spine. Skull base chordomas account for less than 0.2% and chondrosarcomas for less than 0.15% of all intracranial tumors [3]. There is a slight male predominance with a peak incidence between 50 and 60 years of age. The incidence is low below the age of 40 years and very rare in children and adolescents [3-7].

Chordomas are still divided into three subtypes according to the current World Health Organization (WHO) "Classification of Soft Tissues and Bone": conventional, chondroid, and dedifferentiated [4,6]. Using the new criteria in the WHO Tumor Classification, 5th Edition, the pathologic differential diagnoses and biologic behavior of different forms of notochordal tumors were studied. Benign notochordal cell tumor, conventional chordoma, dedifferentiated chordoma, and poorly differentiated chordoma are the four classifications for notochordal tumors [7].

The morphologic subtypes range from the "physaliphorous cells" and myxoid matrix of the conventional type, to matrix that mimics hyaline cartilage in the chondroid type, to biphasic tumors with dedifferentiated chordoma [5]. Chordoma shows many degrees of atypia, yet the relationship between the histological features and the biological behavior remains debatable. Degenerative changes are described in chordomas, though rare, especially in association with radiotherapy or recurrent tumors. These changes include pleomorphism, cellular atypia, necrosis, rhabdoid or epithelioid appearance, and loss of immunoreactivity for S-100, as well as brachyury [4-6].

Typically, chordomas show positive immunoexpression for keratins, T brachyury, S-100, and epithelial membrane antigen (EMA) [3]. The aim of this paper is to assess two case reports of chordoma with cellular atypia, epithelioid appearance, necrosis, and loss of S-100 expression in association with radiotherapy and recurrence. The question is: do these changes correspond to simple post-radiotherapy changes, atypical degenerative changes, or should they be considered as dedifferentiated chordomas?

## Case presentation

Case 1

**Figure 1:**
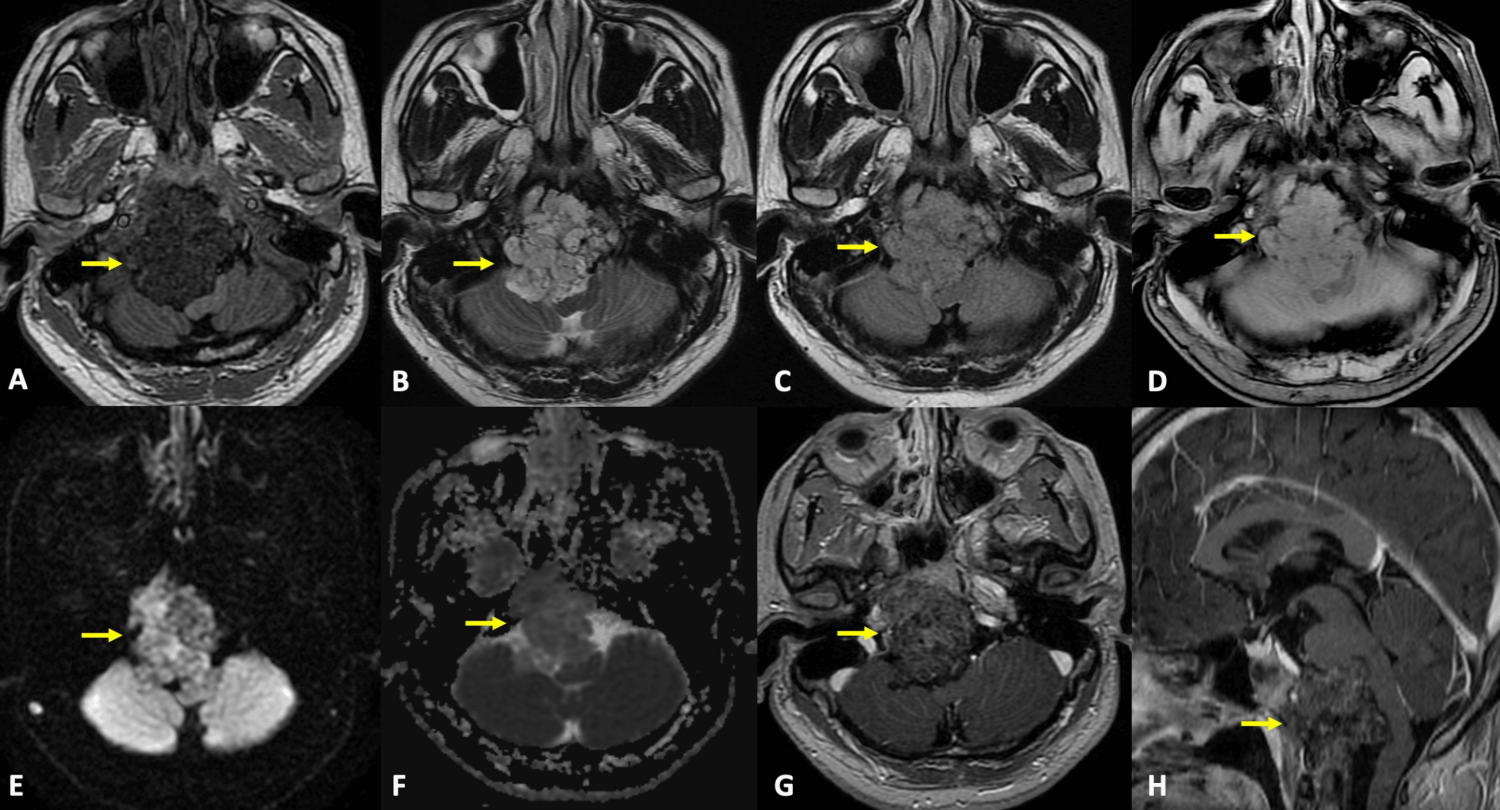
Preoperative MRI with axial and sagittal reconstructions In the clivus region, an irregularly shaped image with partially defined margins and a heterogeneous appearance is observed. On T1-weighted MRI (A), it appears predominantly hypointense compared to the cerebellar gray matter, with linear areas of higher signal intensity. On T2-weighted (B) and fluid-attenuated inversion recovery (FLAIR) (C) sequences, it appears hyperintense with irregular central hypointense lines. The gradient echo sequence (D) shows areas of signal deflection, while the diffusion-weighted sequence (E,F) reveals discrete areas of restricted diffusion. Following contrast administration, there is a heterogeneous enhancement (G,H). This image is associated with erosion of the clivus, right occipital condyle, and petrosal portion of the ipsilateral temporal bone. It extends caudally to contact the odontoid process of the axis and extends superiorly to involve the roof of the nasopharynx and posterior fossa, resulting in compression and displacement of the brainstem (thumb sign). The tumor was marked with a yellow arrow.

**Figure 2:**
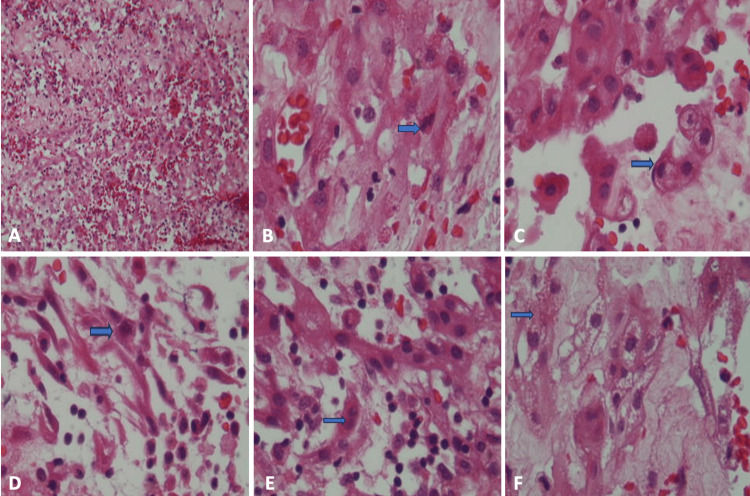
Histological features of Case 1 Histologically, Case 1 reveals a neoplasia composed of large- and medium-sized cells with an elongated appearance in a hemorrhagic stroma (H&E x200) (A). (H&E x400) (B) These cells exhibit cellular atypia and mitotic figures (blue arrow), (C) dense eosinophilic cytoplasm with signet ring cell appearance and nuclei pushed to the periphery (blue arrow), and (D,E) elongated or spindle-shaped cells with double nuclei (blue arrows), (F) and lymphocytic infiltrate (blue arrow). Areas also show alterations in intercellular junctions, fine vacuoles, and dense granules of varying sizes and shapes.

**Figure 3:**
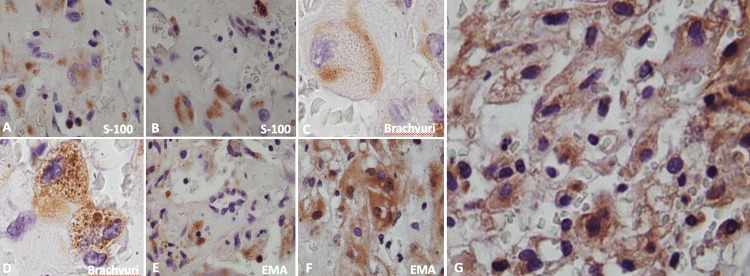
Immunohistochemistry features of Cases 1 and 2 Immunohistochemistry features reveal loss of S-100 expression in both cases (A, B), loss of brachyury expression with granular appearance (C, D), loss of membranal EMA with granular intracytoplasmic appearance (E, F), focal and patchy CDK4 positivity, and cytoplasmic p53 expression in each case (G) (IHQ stain, original magnification x400).

A 33-year-old male, with no relevant medical history, presented with loss of taste, smell, and right hearing. MRI showed a lesion in the clivus compatible with chordoma (Figure 1). Endoscopic endonasal resection of 90% of the lesion was performed. Histologically, it was described as a classic chordoma. The patient received radiotherapy. During follow-up, after five years, there was a recurrence of the lesion. After the new resection, histology identified a neoplasia formed by large- and medium-sized cells, some with an elongated appearance, in a hemorrhagic stroma. These cells showed cellular atypia and mitotic figures. Cells with dense eosinophilic cytoplasm and large vacuoles, resembling signet ring cells, with nuclei pushed to the periphery, were observed. Elongated spindle cells with double nuclei and lymphocytic infiltrate were also noted. In other areas, these cells showed alterations in intercellular junctions, fine vacuoles, and dense granules of variable sizes and shapes (Figure 2). Immunohistochemistry showed positivity for S-100, brachyury, and EMA; however, a loss of immunoreactivity was observed, and Ki67 and p53 were also negative (Figure 3). The diagnosis was chordoma with radionecrosis changes.

Case 2

**Figure 4:**
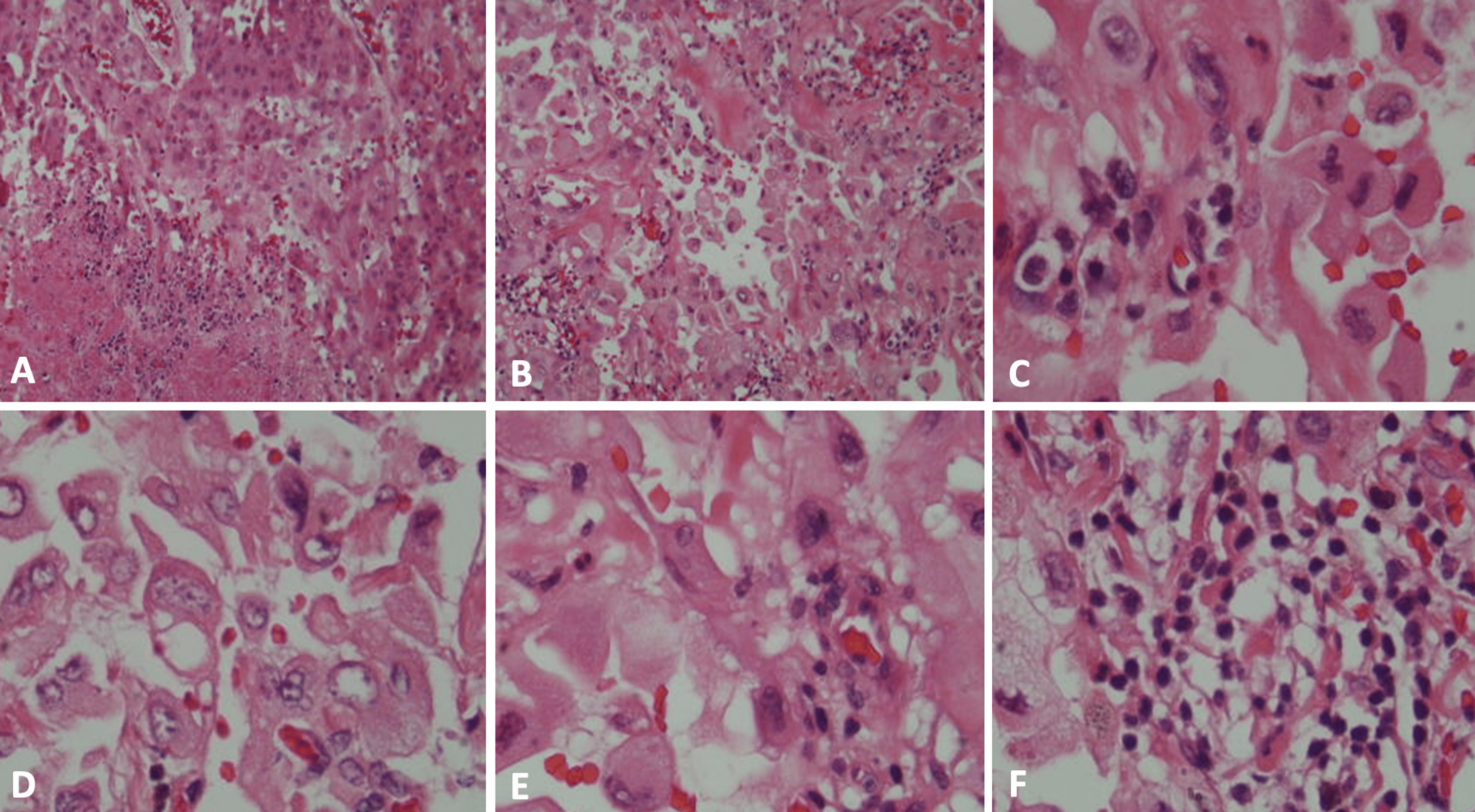
Histological features of Case 2 Histologically, Case 2 shows a neoplasia with large cells in a hemorrhagic background, with foci of fibrosis and numerous blood vessels (H&E x200) (A,B). Cells have an eosinophilic cytoplasm, focal vacuoles (C), nuclei pushed to the periphery creating a signet ring appearance (D), and cellular atypia and irregularities in the cytoplasm (E). Hyperchromatic nuclei, double nucleoli, fusocellular cells, and inflammatory infiltrate and hemosiderophages are also observed (F) (H&E x400).

A 27-year-old male with no relevant medical history presented with headache, vertigo, loss of balance, intense facial pain, and visual disturbances in the left eye, along with numbness and weakness of the arms. An MRI at our institution showed a lesion in the clivus. A surgical resection was performed, and the histological report indicated classic chordoma. He received radiotherapy, and two years later, he was readmitted with intense headaches and visual disturbances. MRI showed a recurrence of the tumor, leading to a new resection of the lesion. Histologically (Figure 4), neoplasia formed by large cells in a hemorrhagic background, with foci of fibrosis and numerous blood vessels, was observed. There was no myxoid stroma. The cells had extensive eosinophilic cytoplasm, displaying small vacuoles and nuclei pushed to the periphery, giving a signet ring appearance. Cellular atypia, hyperchromatic nuclei, double nucleoli, micronucleoli, spindle cells, and inflammatory infiltrate were also noted, along with a loss of staining capacity with routine techniques. Immunohistochemical staining showed that both tumors had a loss of S-100, brachyury, EMA, and CDK4 expression. Ki67 and p53 were negative (Figure 3). The diagnosis was chordoma with changes due to radiation.

## Discussion

Chordomas have three histopathologic subtypes: conventional, chondroid, and dedifferentiated type; conventional chordomas have been reported as the most common subtype, comprising approximately 95% of cases [4]. Histopathologically, conventional chordomas form with fibrous bands or lobulated architecture and a myxoid matrix separating lobules with classic physaliferous cells (PCs).

Chondroid chordomas (CCs) represent a variant that typically arises at the skull base, often displaying overlapping features with conventional chordomas and chondrosarcomas [4,7]. They predominantly localize to the spheno-occipital region of the skull base and account for 5%-15% of cases [6]. CCs show neoplastic cells within lacunar-like spaces embedded in a basophilic matrix resembling neoplastic hyaline cartilage. Cases where the chondroid component predominates can be challenging to distinguish from chondrosarcomas based on morphology alone [3,7].

This bone tumor usually affects children or young adults and is more common in the base of the skull and cervical spine [7]. PDC is typified by sheets of fusocellular or epithelioid cells that are very tiny in size, frequently resembling rhabdoid structures, and devoid of physaliphorous cells [7-9], including variable nucleoli, irregular and oval nuclei, eosinophilic cytoplasm with cytological atypia, elevated cellularity, enhanced mitotic activity, and an unorganized growth pattern. There have been reports of necrosis and inflammation in certain places, but no myxoid or chondromyxoid stroma [3,7,8]. PDC is regarded as a biphasic tumor that typically manifests as high-grade undifferentiated pleomorphic sarcoma or osteosarcoma. It is made up of traditional chordoma with high-grade sarcomatous transformation [8].

Chordomas typically immunohistochemically express positivity for brachyury, cytokeratin, EMA, keratin, and S-100 [4,5]. It is crucial to differentiate them from conventional chondrosarcomas, which are negative for brachyury, cytokeratin, EMA, and positive for S-100 [8,9]. Loss of nuclear INI-1 is generally associated with negative immunoreactivity for S-100, serving as an independent risk factor for tumor recurrence or progression [8,9]. Previous reports have indicated a possible loss of EMA and cytokeratin or brachyury in dedifferentiated chordoma [8,9]. Immunohistochemically, tumor cells are positive for AE1/AE3, EMA, cytokeratin (CK), brachyury, focally S-100P, MNF116, OSCAR, and glypican 3, as per the study by Rekhi [10].

The MIB-1 labeling index is useful in correlating recurrences in chordomas and distinguishing them from ecchordosis physaliphora, with an increased index indicating a higher likelihood of chordoma [8]. Loss of SMARCB1 serves as a hallmark of atypical teratoid/rhabdoid tumors (AR/TT), a rare brain tumor occurring in young children with the deletion of SMARCB1/INI-1 [9]. Recently, aggressive pediatric PDC has been associated with loss of SMARCB1, showing notochordal differentiation and consistent loss of SMARCB1/INI1 [9].

Loss of PTEN and CDKN2A (encoding p16) is one of the most common molecular changes seen in chordomas; poor prognoses are linked to particular inhibitors of the PI3K/AKT/mTOR pathway and CDK4/6. A higher Ki-67 score, a greater potential for metastasis, a shorter overall survival time, and a generally worse prognosis are all correlated with the simultaneous loss of PTEN and p16 [11].

Sarcomatous transformation in chordoma is extremely rare and is recognized as a distinct entity due to its more aggressive clinical course [12-15]. Differentiating between degenerative changes versus malignant transformation as sarcomatous, epithelioid, or rhabdoid differentiation has been reported, primarily occurring after conventional radiotherapy [13-16]. Distinguishing between sarcomatous chordoma and dedifferentiated chordoma remains controversial, especially post-radiotherapy, where proton beam therapy may contribute to tumor dedifferentiation [13,14]. Hara et al. [15] reported a case of sarcomatous transformation after proton beam therapy, occurring three years after initial treatment.

It was reported that, in a series of 52 patients, every single patient treated with either palliative therapy, intralesional intracapsular excision, or radiation alone developed a local recurrence within 17-20 months. By comparison, after en bloc resection with proper margins, only 20% of patients had a local recurrence during 56-94 months. On the other hand, post-radiotherapy recurrences and metastases have also been reported [17]. In a recent retrospective cohort analysis of 25 chordoma patients, Jambhekar et al. [18] discovered a substantial correlation between intratumoral necrosis and spindle cell sarcomatoid characteristics and poor patient outcomes. It remains unclear whether there is a loss or overexpression of immunohistochemical markers in such cases. Positive immunoreactivity for cytokeratin, EMA, S-100, and brachyury has been reported in conventional chordoma foci, while negative expression for p53 was observed. Conversely, positive expression of INI1 and vimentin, with negative or reduced immunoreactivity for cytokeratins (AE1/AE3, CAM5.2), EMA, S-100, and brachyury, has been reported [12,14].

Chordomas also demonstrate dual epithelial-mesenchymal differentiation as they arise from embryonic remnants of the notochord, with brachyury acting as a regulator of notochordal differentiation and a marker for chordoma [10]. Studies have demonstrated that brachyury overexpression is critical for chordoma cell survival and differentiation from chondrosarcomas; nevertheless, despite its great sensitivity and specificity, its relationship to prognosis is still unclear. Overexpression of brachyury causes the epithelial-mesenchymal transition (EMT), a biological process that is reversible in epithelial cells and which improves stem-like characteristics such as motility, invasiveness, and tolerance to common genotoxic chemicals [19].

## Conclusions

Post-radiation changes in chordomas reveal distinct histological alterations depending on the type of radiation used. Pleomorphism, cellular atypia, necrosis, fibrosis, and inflammation are usually a consequence of conventional radiotherapy. Conversely, carbon ion therapy often causes fibrosclerosis and necrosis. These findings suggest that carbon ion therapy may provide effective tumor control with potentially fewer adverse effects compared to conventional radiotherapy. This highlights the potential of carbon ion therapy as a promising treatment for chordomas.
